# 2D materials: a wonderland for physical science

**DOI:** 10.1093/nsr/nwab202

**Published:** 2021-11-26

**Authors:** Yi Xie, Dongyuan Zhao

**Affiliations:** Hefei National Laboratory for Physical Sciences at Microscale, University of Science and Technology of China, China; Department of Chemistry, State Key Laboratory of Molecular Engineering of Polymers, Shanghai Key Laboratory of Molecular Catalysis and Innovative Materials, Laboratory of Advanced Materials, and Collaborative Innovation Center of Chemistry for Energy Materials (iChEM), Fudan University, China

Two-dimensional (2D) functional materials (2DFMs) are materials with 2D features and fascinating physical properties for various applications. The large diameter-length ratio gives them unprecedented property compared to their bulky states. A free surface state, unsaturated active site, flexible light-matter interaction and the strong quantum Hall effect make 2DFMs optimal for many emerging issues. Over the past two decades, tremendous work has been done on the synthesis, characterization and application of 2DFMs. Among this research, seminal work appeared in 2004. In a typical experiment, Geim and Novoselov exfoliated monolayer graphene from bulky graphite, which greatly boosted the enthusiasm for research into 2DFMs [1]. Since then, this subject has become a central issue in physical science and will continue to be a bustling topic in the future. Materials with 2D layered structures, such as hexagonal-boron nitride (h-BN), MoS_2_, metal halides, MXenes, MOFs and COFs, have been synthesized and investigated in a series of domains. These fields cover efficient photonics, gas sensing and separation, antiviral protection and detection, energy conversion and storage, and artificial intelligence.

This special topic aims to highlight the state-of-the-art progress in synthesis and application of 2DFMs and includes two reviews, two research articles and six perspectives. For example, Zhang *et al.* survey the recent advances in wet-chemical synthesis of 2D metal nanomaterials and establish the interdependence of electrocatalytic performance and structure of these 2D catalysts [2]. Besides these intrinsically non-layered metals, 2D van der Waals (vdW) materials are another type of 2DFM. To explicate this point, Liu *et al.* further reviewed emerging hot physical topics and intriguing materials, such as 2D topological materials, piezoelectric materials, ferroelectric materials, magnetic materials and twistronic heterostructures [3]. Synthetic strategies concerning growth mechanisms and preparation conditions, as well as typical examples, are discussed in detail. Finally, prospects and further opportunities in the booming field of 2D materials are addressed.

In one research article, Fu *et al.* introduce their model of a hard-soft-acid-base couple for controlling the 2D nucleation of the predetermined facets and growth direction of crystals, through which a wide variety of high-quality 2D rare-earth oxide single crystals with tailorable facets have been synthesized for the first time [4]. They overcome the bottleneck of thermodynamic stability of different facets. Benefitting from excellent optoelectronic properties, 2D indium selenide in *β*-phase (*β*-InSe) is a promising solution to the problem of application in transistors and photodetectors, and has been widely studied. In the other research article, Zhang and co-authors prepare the stable p-type 2D *β*-InSe via a temperature gradient method [5]. The high photocurrent anisotropic ratio of 0.70 verifies its potential for filter-free polarization-sensitive photodetectors.

Six perspectives introduce the synthesis and application of various 2D materials. Novoselov *et al.* highlight the application of multifunctional 2D materials for antiviral protection and detection, which is a good response to the COVID-19 pandemic [6]. Huang *et al.* further present ultrathin 2D hybrid perovskites for flexible electronics and optoelectronics [7]. Moreover, Zhao *et al.* demonstrate the bottom-up self-assembly approach and its advantage in synthesizing 2D mesoporous materials [8]. With regard to the vdW interactions and atomic-layered nature of 2D atomic crystals (2DACs), Duan *et al.* show how to construct a high-quality heterostructure/superlattice via 2DACs [9]. Dou *et al.* reveal organic semiconductor-incorporated 2D halide perovskites [10]. Inspired by natural enzyme catalysis and to create a globally carbon-neutral society, Sun *et al.* present the emerging 2D materials for solar fuels via artificial photosynthesis [11]. Their wide application further confirms the superiority of 2DFMs in physical science.

From the perspective of basic research, the study of 2DFMs will certainly keep being a hot issue, since the combinations of electronic state and quantum-size effect are good candidates for various subjects. 2DFMs have exhibited the great advantage of creating huge economic benefits in industrial manufacturing. We hope this special topic can promote further understanding of 2DFMs. Last but not least, how to design and precisely synthesize these 2DFMs is a vital but still very challenging step, and this largely restricts their practical application. Some research has shown preliminary signs through integrating advanced techniques. Beyond this progress, one can even foresee an ideal society with 2DFMs as a pillar, containing solutions to carbon neutrality, intelligent information and human health.
